# Magnitude of shift of tumor position as a function of moderated deep inspiration breath-hold: An analysis of pooled data of lung patients with active breath control in image-guided radiotherapy

**DOI:** 10.4103/0971-6203.44475

**Published:** 2008

**Authors:** K. R. Muralidhar, P. Narayana Murthy, D. Shankar Mahadev, K. Subramanyam, G. Sudarshan, A. Krishnam Raju

**Affiliations:** 1 Indo-American Cancer Institute and Research Center, Hyderabad, AP, India; 2 Department of Physics, Nagarjuna University, Guntur, AP, India

**Keywords:** Active breath control, digital reconstruction radiographs, image-guided radiotherapy, iView-GT

## Abstract

The purpose of this study was to evaluate the reproducibility and magnitude of shift of tumor position by using active breathing control and iView-GT for patients with lung cancer with moderate deep-inspiration breath-hold (mDIBH) technique. Eight patients with 10 lung tumors were studied. CT scans were performed in the breath-holding phase. Moderate deep-inspiration breath-hold under spirometer-based monitoring system was used. Few important bony anatomic details were delineated by the radiation oncologist. To evaluate the interbreath-hold reproducibility of the tumor position, we compared the digital reconstruction radiographs (DRRs) from planning system with the DRRs from the iView-GT in the machine room. We measured the shift in x, y, and z directions. The reproducibility was defined as the difference between the bony landmarks from the DRR of the planning system and those from the DRR of the iView-GT. The maximum shift of the tumor position was 3.2 mm, 3.0 mm, and 2.9 mm in the longitudinal, lateral, and vertical directions. In conclusion, the moderated deep-inspiration breath-hold method using a spirometer is feasible, with relatively good reproducibility of the tumor position for image-guided radiotherapy in lung cancers.

## Introduction

Respiratory motion studies have tracked the movement of the tumor the host organ; radiographic fiducial markers imbedded at the tumor site; radioactive tracers targeting the tumor; and surrogate organs, such as the diaphragm, which are assumed to correlate with the tumor.

To reduce the target margin error due to respiratory motion, various respiratory motion-control treatment techniques, including gating,[1–3] breath holding,[4] or active breathing control (ABC),[5,6] are used in radiotherapy. A precise breathing-monitoring system is needed for such techniques. The most commonly used systems are as follows.

The spirometer to measure the change in the lung volume, measurement of the surface of the abdomen by tracking a reflective marker on the chest with a fixed camera[2,7–10] or by measuring the distance from a fixed point to the surface of the abdomen using a laser-based distance sensor[1] and by tracking internal markers using x-ray[11–15] or internal sensors using magnetic fields.[16]

Stereotactic radiotherapy (SRT) in the treatment of lung tumors has been shown to have good results and low morbidity[17,18] and has been supported by various methods using immobilization devices[19] or coordinating systems for respiratory motion. To reduce respiratory motion during Image-guided radiotherapy (IGRT) for lung or liver tumors and improve feasibility for IGRT in elderly patients or patients with pulmonary dysfunction, we used deep-inspiration breath-hold method. Reproducibility of organ position, especially diaphragmatic motion, was verified in healthy volunteers by others.[20] However, the diaphragm position does not necessarily reflect the lung tumor position directly, especially for tumors in the lower lobe, due to the complexity of tumor motion in a clinical setting.[21] Because of this, from June 2007, we have used the ‘2D-2D match analysis of bony landmarks’ method in IGRT for lung tumors and clinically and directly verified the interbreath-hold reproducibility of tumor position using CT scans before the treatment.

## Materials and Methods

### Active breath control system

It consists of the patient's respiratory system, which again consists of mouthpiece, transducer turbine, balloon valve coupler, etc.; mirror support system, patient control switch, RS-232 cable, control computer, and PC extender system transmitter [Figure 1A]. Schematic drawing of the above system is also shown in Figure 1B.

**Figure 1A:**
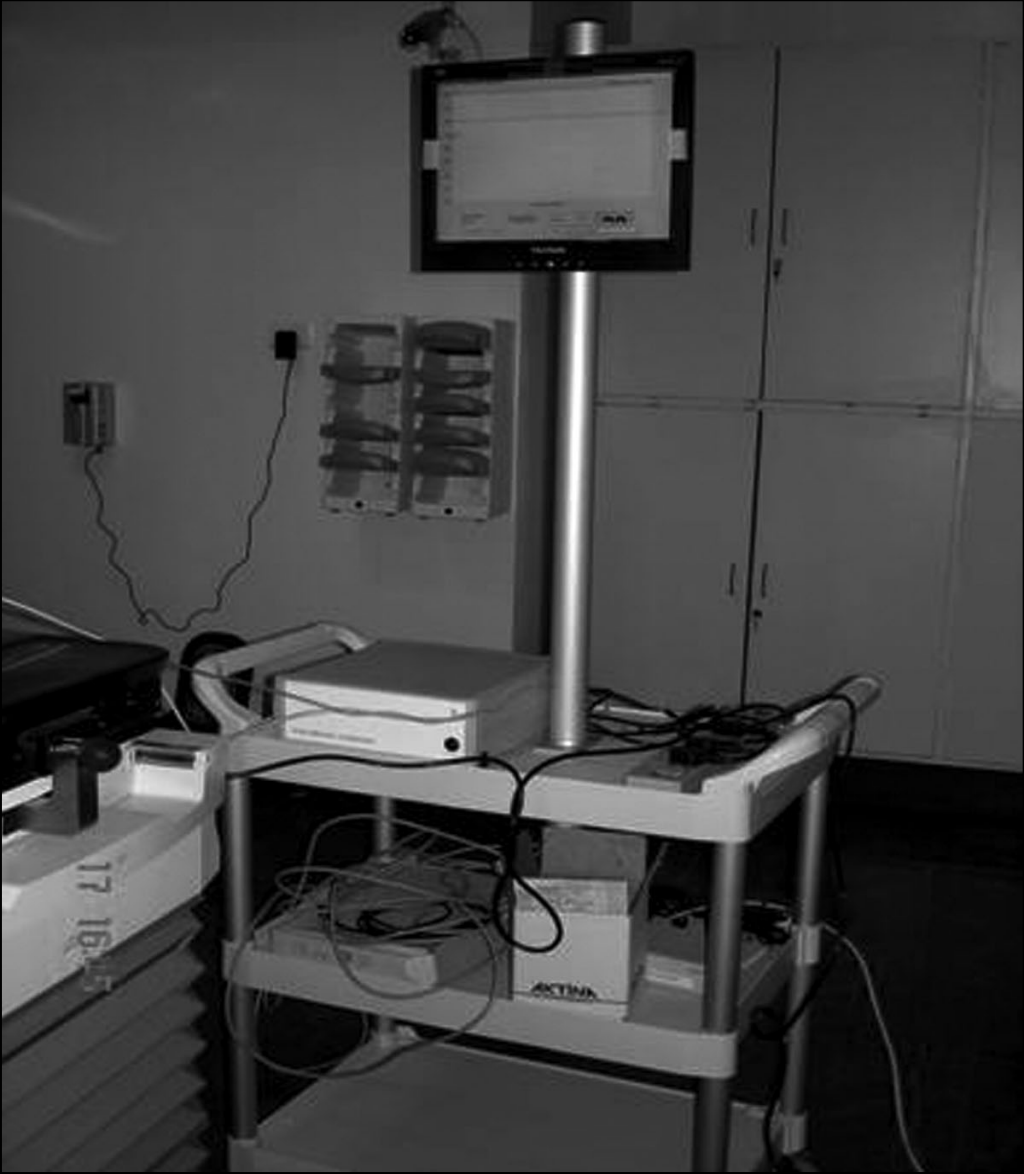
Active breath control system

**Figure 1B:**
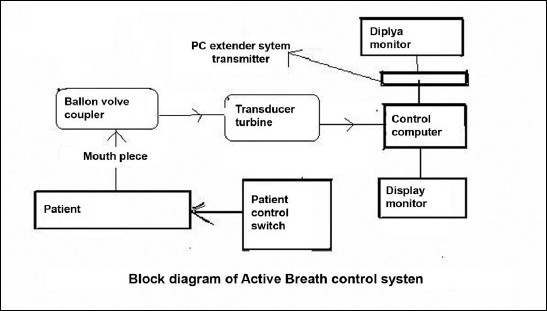
Block diagram of active breath control system

#### Patient background

Eight patients with lung cancer were treated with RT and were studied from March 2007 to January 2008 [Table 1]. All tumors were delivered 5 Gy per fraction at the isocenter, and the total dose was 50 Gy with 10 fractions, with a deep-inspiration breath-hold method using spirometer-based monitoring. The median age was 62 years (range, 47-82 years). Five patients were male and 3 patients were female. Six patients were diagnosed with primary lung cancers, and 2 patients had secondary lymphanode from primary. The median tumor volume was 69 cc (range, 10.33-149.13 cc). Tumor location was as follows: 2 tumors in the right upper lobe (RUL), 2 tumors in the right middle lobe (RML), 4 tumors in the right lower lobe (RLL), and 2 tumors in the left upper lobe (LUL). All tumors were peripheral lung tumors and were not adjacent to other organs, such as the chest wall or heart.

**Table 1:** Patients who underwent IGRT with active breathing control

*Age*	*Gender*	*Tumor size (CC)*
53	M	52.61
65	F	118.49
69	M	149.13
70	M	35.77
55	M	115.7
47	F	10.33
82	M	19.01
55	F	51.03

The deep-inspiration breath-hold method using spirometer-based monitoring in active breath control system

Here we describe the procedure of the active breath control method using a spirometer.

**Figure 2:**
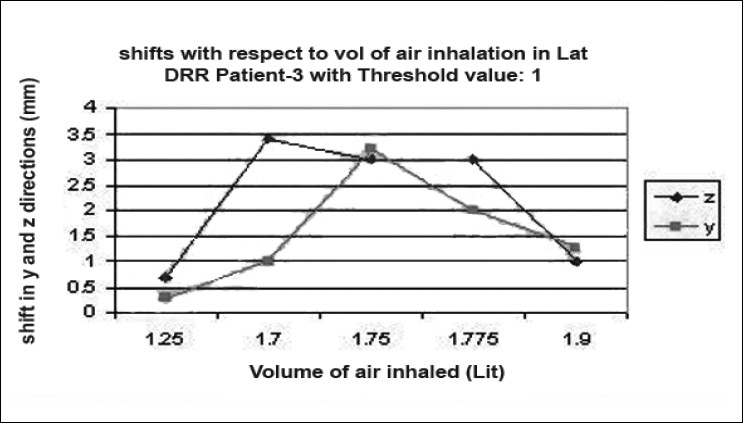
A patient with AIO immobilization. A mouthpiece connected to the spirometer for mDIBH

**Figure 3:**
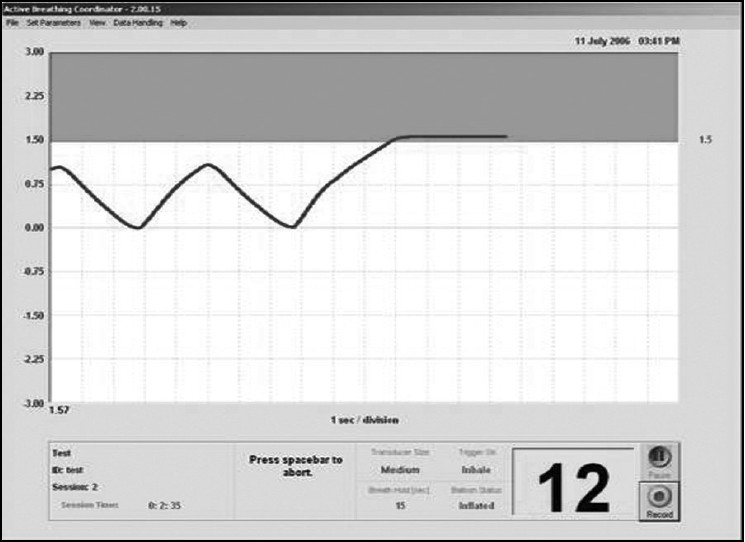
The spirometer-based respiratory motion monitoring system

A reproducible state of maximum breath-hold (deep-inspiration breath-hold [DIBH]) is advantageous for treating thoracic tumors, because it significantly reduces respiratory tumor motion and changes internal anatomy in a way that often protects critical normal tissues. The pneumotach spirometer is a differential pressure transducer that measures air flow; a computer program integrates the signal to obtain the volume of air breathed in and out, which is displayed and recorded as a function of time. To monitor the respiratory phase and breath-holding phase, the patient breathed through a mouthpiece connected to a pneumotachometer. The other end of the pneumotachometer was attached to a spirometer to assist the breath-hold. A nose clip was used to prevent nasal breathing and ensure that the patient breathed through the mouthpiece. For patient setup, we used all-in-one (AIO) immobilization system (POCL, USA) [Figure 2]. The workstation showed the spirometry signal, yielded the respiratory tidal volume of the patient, and was able to display a flow-time curve which can show the state of inspiration, expiration, and breath-hold [Figure 3].

After a predefined volume of air (threshold volume) has passed through the spirometer, a small balloon valve inflates and occludes the tube, applying an assisted BH. The system is configurable to each individual patient or procedure, with variable threshold levels and BH durations possible. At all times, the patient is in control of the BH, via a patient control switch. This switch when depressed will initiate an ABC procedure; and if released, it will automatically open the balloon valve and allow the patient to breathe freely. Before the treatment, patients were familiarized with the procedure with training sessions. For the initial training session, a separate clinic room was used to avoid unnecessary utilization of the CT scanner facility. During this session, explanations were provided regarding the ABC procedure, and an appropriate threshold level and BH duration were determined. Threshold limit was taken as 80% of the maximum value of the deep inspiration. On an average, 20 seconds were taken as time duration for which breath was held. The patient immediately proceeded to the CT scanner, where the 3-D dataset was acquired.

#### CT scan procedure

The largest intra-fraction motion observed is respiratory motion. CT scans (Somatom, Siemens, USA) were performed in the breath-holding (the moderated deep inspiration) phase. Set-up was also performed at the treatment position. Images were acquired with moderate deep-inspiration breath-hold (mDIBH) during 2 to 3 BHs, depending on the BH duration and length of the scan, and were sent to three-dimensional (3D) treatment planning system (CMS XIO planning system, USA). A slice thickness of 2 mm was taken for this study.

#### Target delineation and data analysis

Target delineation was performed on a 3D treatment planning system. A physician delineated the target volume on the axial CT slices using lung CT window settings. Pleural indentations were included within the target. MRI and PET CT were used for delineation.

### Shift verification

**Figure 4:**
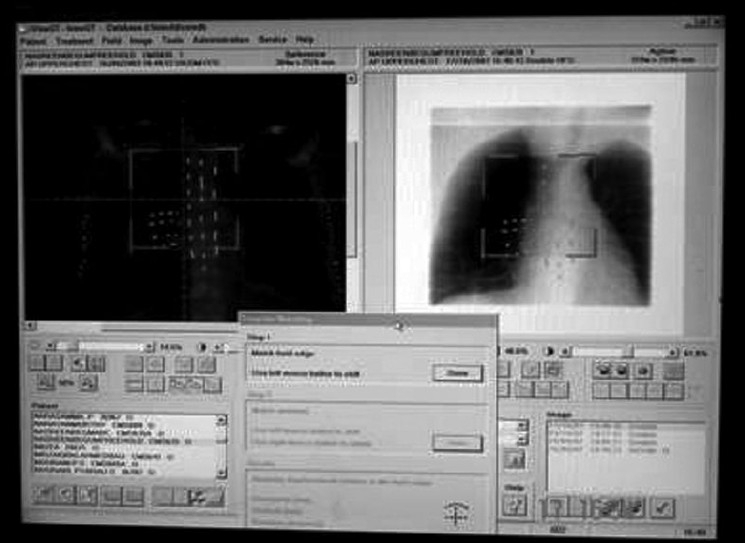
Comparison of DRRs from TPS with iView-GT

**Figure 5:**
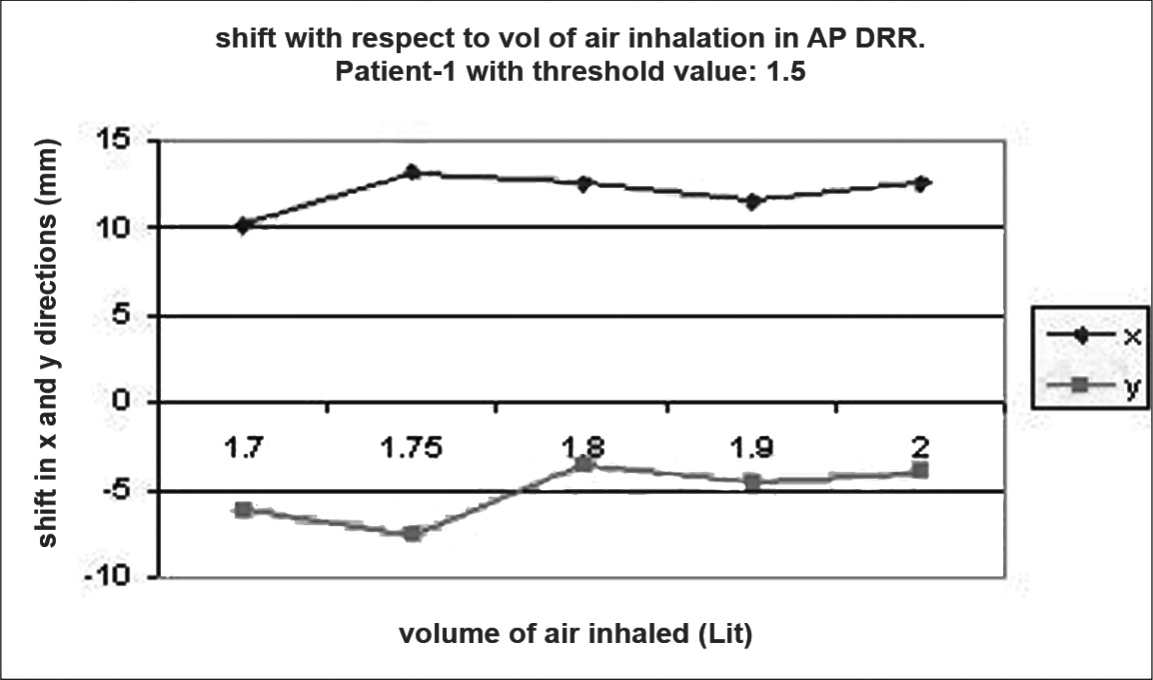
Shift with respect to volume of air inhalation in AP DRR

**Figure 6:**
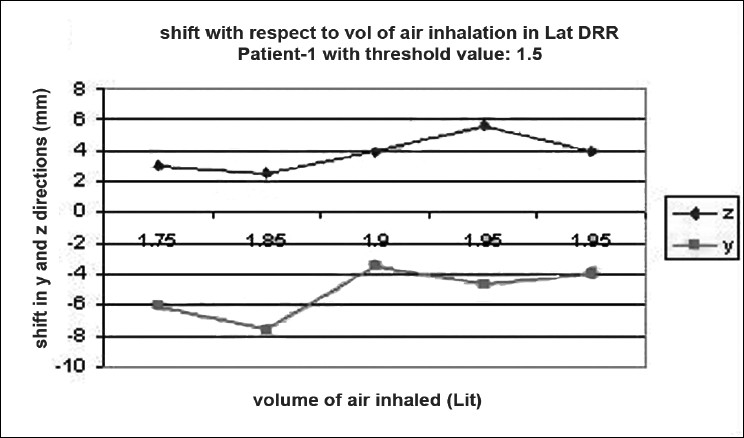
Shift with respect to volume of air inhalation in LAT DRR

**Figure 7:**
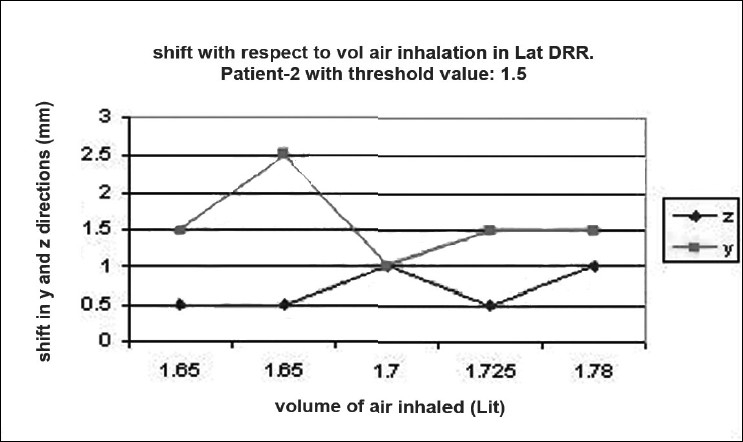
Shift with respect to volume of air inhalation in LAT DRR

**Figure 8:**
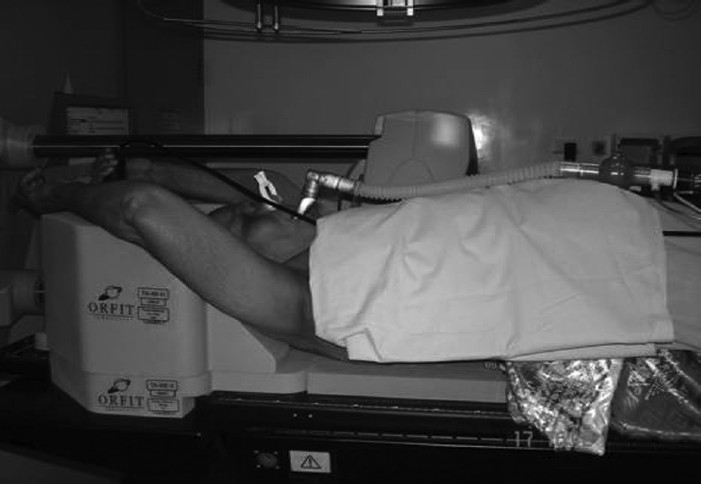
Shift with respect to volume of air inhalation in LAT DRR

**Figure 9:**
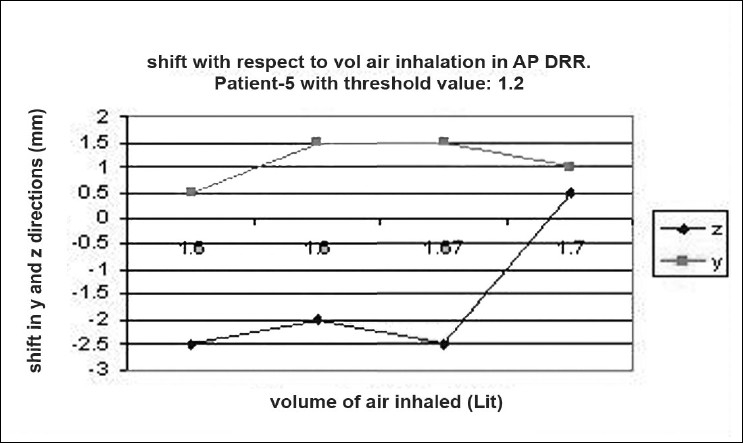
Shift with respect to volume of air inhalation in LAT DRR

**Figure 10:**
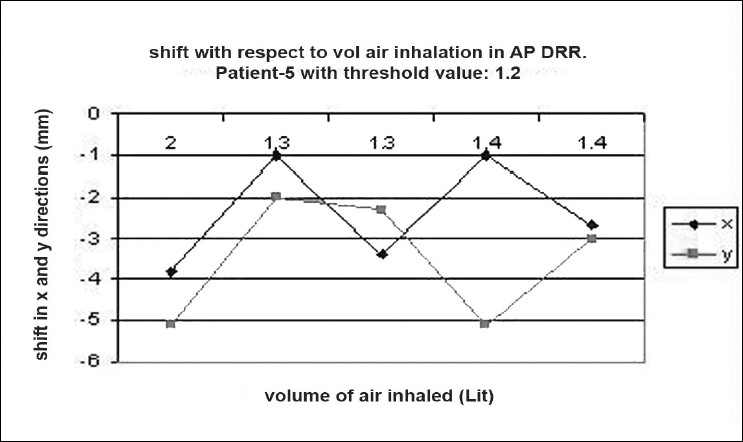
Shift with respect to volume of air inhalation in AP DRR

**Figure 11:**
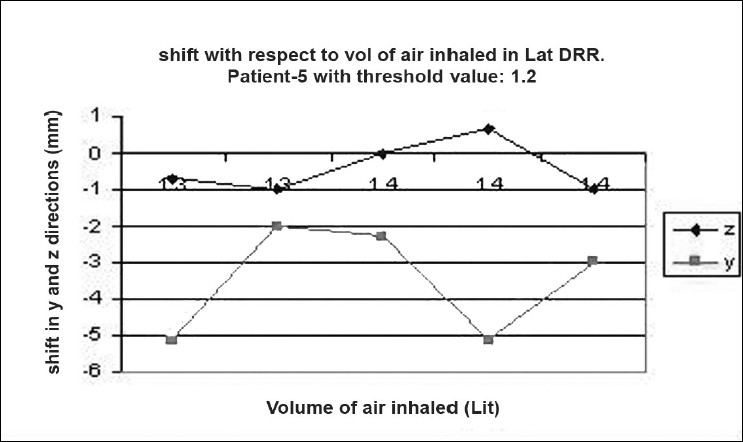
Shift with respect to volume of air inhalation in LAT DRR

Digital reconstructed radiographs from planning system were used to compare the images taken from iView-GT for tumor position and potential motion during ABC in the treatment room using 2D-2D matching tool for bony landmark [Figure 4]. Daily patient position can be corrected based on accurate 4-D data at the time of radiation delivery. Portal images were taken five times with the same threshold that we used for scanning during one treatment delivery. This procedure was chosen to measure the reproducibility that can eliminate any external setup errors, and thus all tumor displacement would be a measure of internal motion only. The deviation was measured and displayed in graphical form, as shown in Figure 5–11. During an ABC procedure, a specifically designed device is utilized to reproducibly apply the same breath-holding level for each session. After a predefined volume of air (threshold volume) has passed through the spirometer, a small balloon valve will inflate and occlude the tube, applying an assisted breath-hold (BH). The system is configurable to each individual patient or procedure, with variable threshold levels and BH durations possible. For the same value of threshold, the shifts in x, y, and z directions of target were noticed and tabulated. This was done with the help of comparison of AP and lateral DRRs with 4D-CT DRRs using 2D-2D match tool in iView-GT. The treatment table was moved to match DRRs, and treatment delivery was begun.

## Results

A total of 80 fractions of radiation were delivered using ABC breath-holds. Our study pertains to 40 observations taken @ 5 mDIBHs per patient in one of the treatment fractions. Up to 3.2 mm intra-fraction motion of the tumor was observed on DRR during ABC breath-holds. The maximum shifts in x, y, and z directions were 3 mm, 3.2 mm, and 2.9 mm respectively, indicating a change of tumor position over the course of treatment with breath-holds at the same phase of the respiratory cycle (mDIBH) with the same value of threshold.

## Discussion

Good reproducibility of the tumor position has been reported in all kinds of breath-hold methods. Cheung *et al.*[22] reported that the inter-fraction reproducibility of the tumor position with the active breath control (ABC) device, which temporary immobilizes the patient's breathing and the average displacement of the GTV center was 0.3 mm (±1.8 mm), 1.2 mm (±2.3 mm), and 1.1 mm (±3.5 mm) in LR, AP, and CC directions respectively.

According to Cheung *et al.*, the average (±standard deviation) displacement of GTV centers with ABC breath-hold applied was 0.3 mm ((±1.8 mm), 1.2 mm ((±2.3 mm), and 1.1 mm ((±3.5 mm) in the lateral direction, anterior-posterior direction, and superior-inferior direction respectively.[23]

The DIBH maneuver was found to be highly reproducible, with intrabreath-hold reproducibility of 1.0 ((±0.9) mm and interbreath-hold reproducibility of 2.5 ((±1.6) mm, as determined from diaphragm position. Patients were able to perform 10 to 13 breath-holds in one session, with comfortable breath-hold duration of 12 to 16 seconds.[24]

In our study, the shift in the tumor position was 3.2 mm, 3.0 mm, and 2.9 mm in CC, LR, and AP directions respectively by using the moderated deep-inspiration technique by matching bony anatomy from TPS to iView-GT-through DRRs. To derive the shift values, a model table for a single patient out of 10 patients is shown in Table 2. These results are similar to the results of other reports with breath-hold methods. Seppenwoolde *et al.*[21] demonstrated that the trajectory of the tumor during inhalation is different from the trajectory during exhalation, i.e., hysteresis, by analyzing the 3D motion of lung tumors during radiotherapy using a real-time tumor-tracking system, and suggested the complexity of tumor motion, especially that of tumor motion in the lower lobe. Our study suggested that our method would be effective for temporary immobilization of respiratory motion in the lower lobe and in the upper lobe.

**Table 2:** Deviations in x, y, and z directions for patient-1

*Threshold*	*Volume of air Inhalation (Liters)*	*x*	*y*	*z*
1.5	1.75	13.2	−7.6	2.7
1.5	1.8	12.6	−3.5	3
1.5	1.9	11.6	−4.6	4
1.5	2	12.6	−4.8	5.6

Maximum deviation (mm) 1.6, 3.0, 2.9

We have to pay more attention to the patient-training sessions and monitoring of respiratory motion during treatment. The advantage of our method is feasibility in many patients and adaptability by many institutions because patients are able to hold their breath more comfortably at the deep-inspiration phase. One more advantage of this study is single-time CT with ABC is sufficient instead of taking CT scan 4 to 5 times to study the intrafraction movement of the tumor. In our experience, this method is very much feasible for elderly patients also.

We calculated geometric uncertainties of the tumor position from our results. There have been many reports focused on the geometric uncertainties of the setup.[25–28] However, there are few reports focused on the geometric uncertainties of internal organs or tumor motion.

Stroom *et al.* and Kutcher *et al.* calculated the geometric uncertainties in radiotherapy due to internal organ motion and external setup deviations. Both deviations consist of a systematic component, which is the same for each fraction of the treatment; as well as a random component, which varies from day to day.[29,30]

Stroom *et al.*[29] calculated the geometric uncertainties of lung cancer patients. In this study, systematic deviations were 2 mm, 3 mm, and 3 mm in LR, AP, and CC directions respectively, and random deviations were 4 mm, 5 mm, and 5 mm in LR, AP, and CC directions respectively. However, it was considered meaningful to obtain the reproducibility of the tumor position (x, y, z vectors), as well as systematic and random motion deviations by our breath-hold method; and we believe that the internal margin of the interbreath-hold reproducibility of lung tumor position would be within almost 5 mm in vector for the treatment planning of IGRT or SRT due to the relatively good reproducibility of our method.

## Conclusion

Even though we are using very sophisticated instruments like active breathe control systems to control the moving targets, there are still some intrafraction variations in the position of the tumor. Daily tumor targeting of lung cancers is feasible with the help of DRRs from planning system when compared with DRRs from iView-GT. Much accuracy is needed when we go for hypo-fractionation. Due to these reasons, we studied the intrafraction variations by using mDIBH in a single fraction 5 times on 8 patients. In our study, we have taken the DRRs of the bony anatomy as reference. We compared the DRRs from the TPS to the DRRs from the iView-GT in selected gating phase. Our data demonstrate good intrafraction reproducibility of lung tumor position using ABC with same value of threshold limit.

A patient cannot inhale the same amount of air every time even though the threshold value is kept the same. As the volume of inhalation is different for different durations, the maximum shift observed is less than 4 mm. For maximum difference of inhaling volume, there is a need for observing the shift. The results that were obtained were closer with other authors' results that compared TPS data to the iView-GT data by using soft tissues, external markers. and internal permanent markers. If this shift is less than the permissible level, we can continue treatment; otherwise, new session of mDIBH should start. Also, a margin of 5 mm has to be given while contouring itself for compensating the magnitude of the shift of tumor position with respect to mDIBH. This procedure will enable us to provide better and more accurate treatment.

Finally, we can conclude that the moderated deep-inspiration breath-hold method using a spirometer is feasible, with relatively good reproducibility of the tumor position for image-guided radiotherapy in lung cancers.
